# Next-generation cardiac pump improves outcomes, cuts costs

**Published:** 2018

In a presentation at the annual meeting of the Heart Failure Association of the European Society of Cardiology, investigators from Brigham and Women’s Hospital recently presented evidence that a next–generation cardiac pump device not only improves long–term outcomes but may also decrease cost of care over time for heart failure patients.

The research team analysed results from the MOMENTUM 3 trial, which compared two devices: the HeartMate II (current generation) and HeartMate 3 (a novel, centrifugal–flow pump), both manufactured by Abbott Inc, which sponsored the study.

‘The HeartMate 3 left–ventricular assist device (LVAD) is a more forgiving pump in terms of clinical adverse events, and now we can confirm that its increased effectiveness is associated with decreased costs,’ said Dr Mandeep Mehra, executive director of the Centre for Advanced Heart Disease and medical director of the Heart & Vascular Centre at Brigham and Women’s Hospital. ‘In medicine, most often, a therapy that demonstrates increased effectiveness usually comes at a higher price, and we are able to show that this new technology actually decreases costs to payers and patients over time.’

Mehra and colleagues found that the newer device reduced costs due to re–hospitalisation by 51%, largely driven by a decrease in stroke and pump malfunction requiring re–operation due to pump thrombosis. Patients who received the HeartMate 3 experienced fewer hospitalisations and, on average, spent 8.3 fewer days in the hospital per year than those who received the HeartMate II. The authors note that it may be possible to further reduce costs by decreasing outlier complications and reducing hospital length of stay and decrease early complications by improving patient selection criteria and considering this therapy before patients get too sick.

In April, Abbott Inc issued a field safety notice regarding HeartMate 3 outflow graft twist complications with an incidence rate of 0.72%. The US Food and Drug Administration (FDA) issued a Class I recall but did not recommend the return of LVADs or avoidance of use in new patients. The current study re–reviewed 20 hospitalisations (five in the HeartMate 3 and 15 in the HeartMate II populations) and in a conservative analysis, classified them as being device–related for the purposes of this analysis. Nonetheless, the data still demonstrated a reduction in re–hospitalisation–related hospital days and significant cost savings for the HeartMate 3 compared to the HeartMate II.
